# Prevalence and associated factors of anxiety and depression among women seeking digital mental health services in Bangladesh

**DOI:** 10.1371/journal.pmen.0000715

**Published:** 2026-09-15

**Authors:** Kamrun Nahar Koly, Jobaida Saba, Fahmida Sarker, Elma Rudhsarah, Md Arif Billah, Rasma Muzaffar, Nafisa Mosaddek, Abu Syed Md. Mosaddek, Daniel D. Reidpath

**Affiliations:** 1 Women Support Initiative Forum-WSIF, Dhaka, Bangladesh; 2 Department of Public Health and Informatics, Jahangirnagar University, Dhaka, Bangladesh; 3 Research Unit of Population Health, Faculty of Medicine, University of Oulu, Oulu, Finland; 4 Department of Public Health, Asian University for Women, Chittagong, Bangladesh; 5 Urban Health Systems and Population Studies Division, icddr, b, Dhaka, Bangladesh; 6 Department of Public Health, School of Health & Life Sciences, North South University, Dhaka, Bangladesh; 7 Department of Environmental Science, Bangladesh University of Professionals, Dhaka, Bangladesh; 8 Department of Pharmacology, Uttara Adhunik Medical College, Dhaka, Bangladesh; 9 Institute for Global Health and Development Division, Queen Margaret University, Edinburgh, Scotland, United Kingdom; Instituto Federal do Maranhão: Instituto Federal de Educacao Ciencia e Tecnologia do Maranhão, BRAZIL

## Abstract

Online mental health services (MH) are playing a pivotal role by increasing MH literacy, enabling accessibility, and reducing logistical barriers, especially for women from developing nations like Bangladesh. However, the psychological burden and service needs of women using online MH platforms remain understudied despite their heightened vulnerability. Therefore, this study investigated the prevalence and determinants of anxiety and depression among women who received counseling from the online MH service platform,Women Support Initiative Forum (WSIF)through a cross-sectional study conducted between 5th September 2023 and 26th August, 2024. Data were collected by WSIF psychologists using a structured client information form covering socio-demographics, history of MH condition, health conditions, and presenting MH symptoms. Anxiety and depression levels were assessed using the Generalized Anxiety Disorder-7 (GAD-7) and Patient Health Questionnaire-9 (PHQ-9). Multivariate logistic regression was used to identify associated factors. Out of 375 women, 59.2% and 58.1% had experienced mild to severe anxiety and depression, respectively, with 25.6% having both conditions. Women with graduate-level education had 66% lower, and those with one or more siblings had 50–54% lower likelihoods of anxiety compared with women without graduate-level education and siblings. In contrast, women with graduate-level or postgraduate-level education and reporting hormonal conditions had 3.41 times, 2.63 times and over 10 times higher likelihoods of depression, respectively, compared with women who were not graduates and without a hormonal condition. Conversely, women with a history of MH and reported migraine, headache and allergic conditions had 52% and 65% lower odds compared with women without these conditions. Distress, negative thinking, and feelings of worry were highly prevalent among women with anxiety and among those with depression. Our findings highlight a significant MH burden among women who accessed online psychological counseling, indicating the importance of targeted actions in designing MH interventions by relevant stakeholders and policymakers.

## Introduction

Globally, mental health (MH) conditions significantly influence people’s quality of life, productivity, and social relationships, accounting for roughly 7% of all disability adjusted life years (DALYs) and 19% of years lived with disability (YLDs) reported by the World Health Organization (WHO) [1,2]. In South Asia, the pooled prevalence of the most common mental health conditions, such as depression and anxiety, is 26.4% and 25.8%, respectively, and most studies identified that women are the most vulnerable [3,4]. A similar situation is observed in Bangladesh, with a nationwide study showing the burden of these conditions is higher among women (21.5% women vs. 15.7% men); however, 90% of women with MH conditions do not seek any form of mental healthcare, reflecting a severe treatment gap [5–9]. However, there is a lack of evidence in Bangladesh and globally that discusses the burden of depression and anxiety among women who can access online MH care.

Recent studies have shown that depression and anxiety account for a significant global health burden even among socioeconomically advantaged women. In India and Nepal, women from urban and higher socio-economic status revealed a substantial burden of depression (22% to 26%) and anxiety (23% to 37%), with associated factors such as age, geographical areas, level of education, household wealth, domestic stress, occupational pressure, and marital status [10–12]. A similar pattern emerged among 30–45% of women in Pakistan, Nigeria, Brazil, and Mexico, and was often linked to marital dissatisfaction, work status, and socio-cultural expectations [13–16]. Even within health facility-based or tele psychiatry-based studies conducted in Pakistan, Nepal, SriLanka, and Ethiopia, a high burden of depression, ranging from 8.6%-41.1%, and anxiety, 13.3%-46.7%, was found among the mental health service seekers, with common associated factors such as being female, being married, having a larger family size, unemployment and low educational attainment [17–19]. These studies intersect across income, education, and social strata and represent a growing yet under-recognized component of the global mental health burden.

As a developing country, Bangladesh faces severe structural inadequacies (only 0.2 psychiatrists and 0.3 psychologists per 100,000 population), which constrain MH service availability and limit center services in the capital city serving 162 million people [20–23]. Additionally, the exposure and interaction between different factors such as gender-based violence and discrimination, economic insecurities, socio-cultural expectations, household stress, restricted decision-making, and reproductive health changes can further deteriorate the mental health state and motivation for care-seeking of women living in resource-constrained settings like Bangladesh [20–29]. In light of these challenges, digital MH services offer a compelling alternative, addressing critical barriers such as stigma and limited mobility, particularly for women, by providing confidential, flexible, and accessible psycho-social support [30–34]. Within this framework, the Women Support Initiative Forum (WSIF) has been established as an innovative Facebook-based platform that delivers tailored digital MH services and establishes a peer support network for women, thereby facilitating secure, convenient access to the non-judgemental and empathetic mental health professionals across the nation [35–37].

Although research on digital MH services has expanded over the years, there remains limited understanding of the characteristics and MH status of women who use such support in the developing country context [30,33,37–39]. Addressing this gap is critical in the Bangladeshi context to tailor gender-specific care, reduce inequities, and improve the effectiveness of available online MH services. Thus, this study explored the prevalence and determinants of depression and anxiety among women who utilized online MH services from WSIF.

## Methodology

### Ethics statement

The study was approved by the Ethical Review Committee of Uttara Adhunik Medical College, Bangladesh (Ref: UAMC/ERC/10/2021/28). Permission to access and use anonymized client records for research purposes was also obtained from the Women Support Initiative Forum (WSIF). As counseling services were delivered online, verbal informed consent was obtained and documented by the attending psychologist before any study-related information was recorded. Participants were informed about the voluntary nature of participation, confidentiality procedures, their right to withdraw at any time without affecting the counseling services they received, and the potential use of anonymized data for research purposes. To protect participants privacy while discussing sensitive mental health issues, counseling sessions were not audio- or video-recorded.

### Study design and setting

This cross-sectional quantitative study was conducted among Bangladeshi women who accessed online MH counseling services from the WSIF between 5th September 2023 and 26th August 2024 [30,33]. WSIF is a women-led online platform founded as a research translation project in 2018, with financial support from the Edward M. Kennedy (EMK) Centre. WSIF aimed to increase mental health literacy and psycho-social well-being among Bangladeshi women. Since its inception, WSIF has provided over 2,700 individual MH counseling sessions, 35 public MH awareness sessions (both online and offline), and peer support via moderated closed Facebook groups for over 70,000 women.

WSIF’ s psychological support team consists of expert psychologists and psychiatrists trained at top public universities in Bangladesh. These professionals have been practicing in both clinical, educational and counseling psychology departments for the past 6–10 years. They provide support for a range of mental health concerns, including anxiety, depression, stress-related disorders, trauma-related conditions, and other psychological difficulties. Mental health service providers select interventions and tailor them to individual client needs and to the psychologist’s expertise. Common approaches include psycho-education, cognitive behavioral therapy (CBT), tele-counseling, transactional analysis, eye movement desensitization and reprocessing (EMDR), neuro-linguistic programming (NLP), supportive counseling, and other evidence-informed psychological interventions.

As part of routine care, on the first day of the session with a client, the psychologist conducted rapport-building and completed the structured Client Information Form (CIF). The CIF collected information on the participant’s age, education, occupation, marital status, living location, history of mental illness, family history of mental illness, number of siblings, self-reported health conditions and presenting MH symptoms. The CIF was developed based on a review of the relevant literature, further refined through discussions among the WSIF team and multiple consultations with experienced mental health professionals to ensure its’ contextual and situational relevance for women seeking digital mental health services in Bangladesh [38–42].

Additionally, psychologists administered two validated psychological assessment tools: the Generalized Anxiety Disorder-7 (GAD-7) scale and the Patient Health Questionnaire-9 (PHQ-9), which were used to assess anxiety and depressive symptoms, respectively. During the first counseling session, attending psychologists documented participants’ presenting mental health symptoms in the CIF based on participants presenting complaints and clinical assessment. For data analysis, the recorded symptoms were subsequently reviewed and coded by a senior WSIF psychologist (to ensure consistency in symptom classification) into five broader categories: stress-related symptoms, negative thinking, trauma-related symptoms, feelings of worry, and self-harm according to the fifth edition of the Diagnostic and Statistical Manual of Mental Disorders (DSM-5) [43]. The individual symptoms included within each category are provided in Table 1. These symptom categories were analyzed independently of the GAD-7 and PHQ-9 assessments. Information regarding therapeutic approaches and recommendations provided during counseling sessions was also documented in routine client records. However, WSIF does not maintain narrative counseling transcripts, audio or video recordings, or detailed textual counseling notes. Consequently, no qualitative data were extracted or analyzed for this study.

**Table 1:** Presenting symptoms of study participants.

*Categories*	*Presenting symptoms*
*Stress-related symptoms*	Restlessness, Irritability, Anger issues, Difficulties in concentration, Decision-making issues
*Negative thinking*	Sadness, Loneliness, Emotional imbalance, Changes in sleep activity, Coping with daily issues, Relationship issues, Attachment issues, Feeling down, Low self-esteem, Negative thoughts, Lack of interest, Insecurities
*Trauma-related symptoms*	Trauma, Adverse childhood experience
*Feelings of worry*	Anxiousness, Overthinking
*Self-harm*	Self-harm behavior

### Study participants

**Fig 1:**
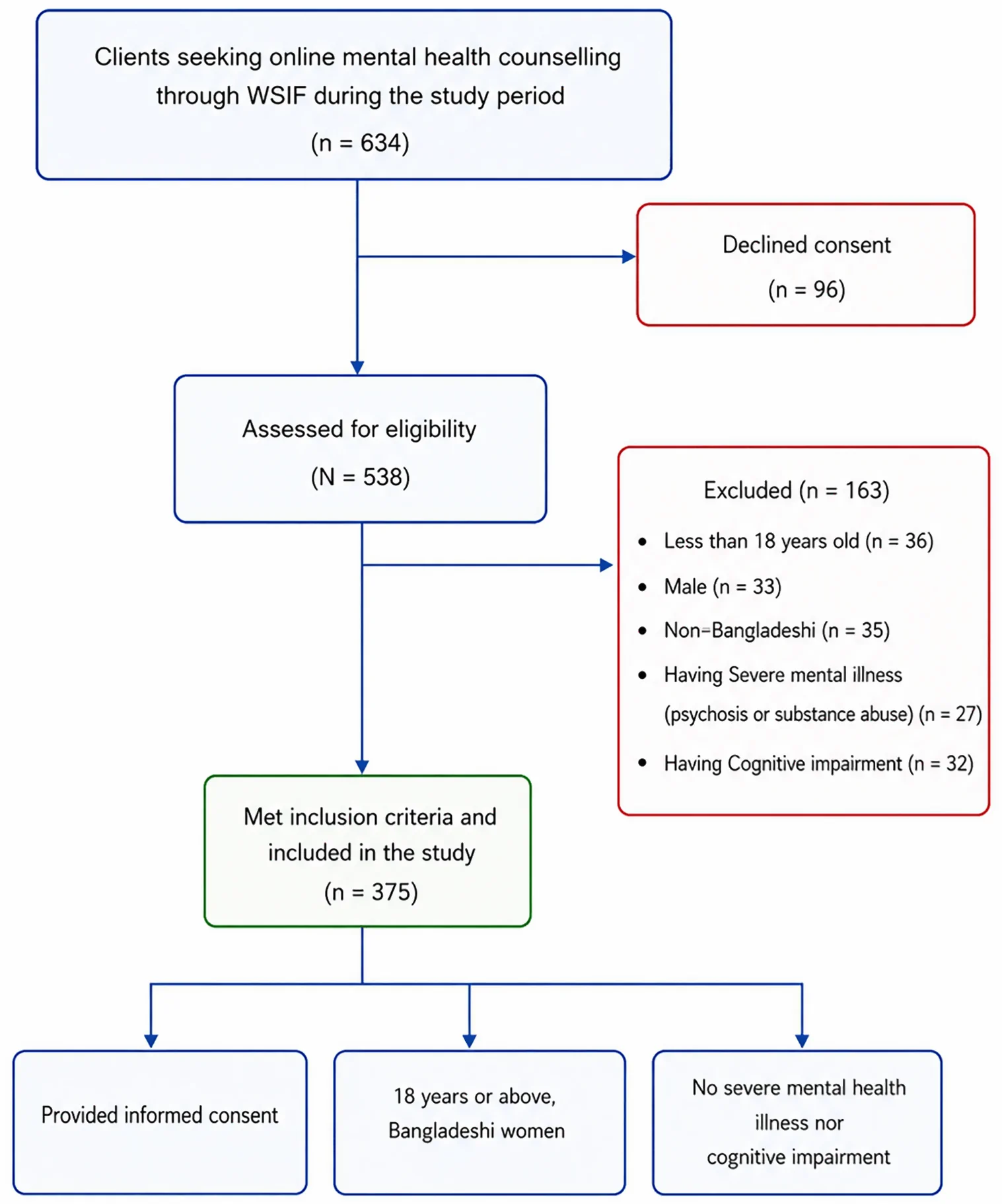
Flow diagram of participant selection, eligibility assessment, and inclusion in the study.

Within the study timeline, 634 clients sought online counseling services from WSIF; however, only 375 women met the inclusion criteria of (i) being self-identified and aged 18 years or above, (ii) being Bangladeshi nationals, (iii) willingness to partake in the study and provide informed consent (Fig 1). Information on biological sex and gender identity was not collected separately. The reasons for exclusion were that women with severe MH conditions, including substance abuse or psychosis, as well as those with cognitive impairments, were excluded from the study.

### Data collection

The research team retrospectively extracted anonymized data from the WSIF digital database, which recorded all CIF data from 1^st^ November 2024–31^st^ December 2024, where all clients’ mental health records were securely stored in their encrypted digital patient record system. The data analysts and authors had no identifiable information about participants. During counseling sessions, WSIF psychologists informed their clients about their voluntary use of anonymized data for research purposes. Their willingness was also documented throughout the process, and confidentiality was strictly upheld. The team extracted information from those CIFs and entered it into a separate analytical database, collecting clients’ data using a CIF during their first counseling sessions. Given the platform’s therapeutic nature, data collection focused on minimal levels of personal, medical, and family-related information to maintain participant confidentiality and ethical research standards.

CIF documented key socio-demographic and health-related variables scientifically known to influence MH outcomes: age, education, occupation, marital status, living location [38], history of mental illness [39], family history of mental illness [40], number of siblings [41], health conditions [42], and presenting MH symptoms. Psychological assessment tools included the Generalized Anxiety Disorder-7 (GAD-7) scale and the Patient Health Questionnaire-9 (PHQ-9) for assessing the levels of anxiety and depression in participants, respectively. Additionally, women’s presenting MH symptoms were documented as descriptive, session-based observations and were not derived directly from the GAD-7 or PHQ-9 items.

### Study variables

#### Dependent variables.

The dependent variables were the levels of anxiety and depression, assessed by the GAD-7 and the PHQ-9 scales.

The GAD-7 scale consists of 7 items, and the PHQ-9 scale consists of 9 items; both scales have been validated and used in several studies in Bangladesh [44–49]. The psychologists asked participants about each item over the past two weeks, and overall scores were generated for each participant. We extracted the GAD-7 and PHQ-9 scores, and the symptom severity scores were coded as 0–4 for minimal/none, 5–9 for mild, 10–14 for moderate, and 15–21/27 for severe levels of anxiety and depression [45,49–51]. The standard PHQ-9 interpretation was slightly modified, combining the moderately severe [15–19] and severe [20–27] categories into a single severe category for analysis. We recoded the GAD-7 and PHQ-9 scores, ranging from 0 (no) to 1 (mild to severe) for analytical purposes. The internal consistency of the GAD-7 and PHQ-9 was reported as Cronbach’s α  = 0.7536 and 0.7012, respectively.

#### Independent variables.

In this study, independent variables included women’s age groups (>25, 25-29, 30-34, 35-39 and 40+), educational level (not graduate, graduate, and postgraduate) [42], occupations (housewife, student, job holder and business), marital status (never married, currently married, and formerly married) and living location (within Dhaka, outside of Dhaka, and outside of Bangladesh), number of siblings (only child, 1–2 siblings, and 3 + siblings) and a history of MH conditions for both personal and familial (yes/no). Additionally, the psychologists documented and assessed women’s presenting MH symptoms during the first counseling session. Participants’ self-reported health conditions were coded into morbidity categories, including chronic, gynecological, hormonal, and other illnesses, to ensure standardized classification (S1 Table).

#### Statistical analysis.

General characteristics of the study participants were presented using frequency distributions for categorical and dichotomous variables and descriptive statistics (mean and standard deviation (SD)) for continuous variables. When necessary, variables were recategorized for frequency analysis. In our study, each participant was included only once in the datasets, and all analyses were performed at the individual participant level, ensuring that no participant was counted more than once. Levels of anxiety and depression were evaluated using the percentage distribution with a 95% confidence interval (CI). Finally, to identify factors associated with anxiety and depression, separate multivariable logistic regression models were used. Adjusted odds ratios (AORs) with standard errors (SEs), 95% CIs, and p-values were reported. Each AOR therefore represents the association between the predictor and outcome while controlling for all other covariate in the model. Separate multivariable logistic regression models were constructed. All covariate of interest (age, education, occupation, marital status, history of MH conditions, family history of MH conditions, number of siblings, living location, and medical history) were entered simultaneously into each model. Adjusted odds ratios (AORs) with standard errors (SEs), 95% CIs, and p-values were reported. Each AOR therefore represents the association between the predictor and outcome while controlling for all other covariate in the model. The overall analysis in the study was conducted in Stata Windows version 15.2 (Stata Corp, TX). This study adhered to the Strengthening the Reporting of Observational studies in Epidemiology (STROBE) Guidelines for observational studies(S1 Checklist)**.**

## Results

### General characteristics

The mean age of the 375 female participants was 29.89 years (SD ± 7.21), with the majority of service clients aged 25–34. In terms of educational attainment, approximately 87.5% were at least graduates. The majority (52.27%) were employed, while 26.4% were students, and 18.1% were housewives. More than half (57.1%) of the participants were married at the time of the study, followed by 37.1% who had never been married. 58.4% of participants reported having 1 or 2 siblings, 26.7% had 3 or more siblings, and 14.9% were the only child. Most of the respondents (67.2%) were from Dhaka, 18.4% lived outside Dhaka, and 14.4% accessed the services from abroad. Among the participants, 23.8% reported a prior medical condition, including chronic illnesses, gynecological conditions, hormonal illness, and other illnesses. Additionally, approximately 19.7% had a history of MH conditions, and approximately 9.33% reported a family history of MH conditions (Table 2).

**Table 2:** General characteristics of the study population (n = 375).

*General characteristics*	*n*	*%*	*Mean (SD)*
***Age***			**29.89 (7.21)**
*<25 years*	67	17.87	
*25-29 years*	135	36.00	
*30-34 years*	118	31.47	
*35-39 years*	30	8.00	
*40 + years*	25	6.67	
***Education***			
*Not graduate*	47	12.53	
*Graduate*	154	41.07	
*Postgraduate*	174	46.40	
***Occupation***			
*Housewife*	68	18.13	
*Student*	99	26.40	
*Job holder*	196	52.27	
*Business*	12	3.20	
***Marital status***			
*Never married*	139	37.07	
*Currently married*	214	57.07	
*Formerly married*	22	5.87	
***Types of health conditions***			
***Chronic***			
*No*	349	93	
*Yes*	26	7	
***Gynecological***			
*No*	363	96.8	
*Yes*	12	3.2	
***Hormonal***			
*No*	367	97.9	
*Yes*	8	2.1	
***Others***			
*No*	332	88.5	
*Yes*	43	11.5	
***History of MH conditions***			
*No*	301	80.27	
*Yes*	74	19.73	
***Family history of MH conditions***			
*No*	340	90.67	
*Yes*	35	9.33	
***Number of siblings***			**1.84 (1.18)**
*Only child*	56	14.93	
*1-2 siblings*	219	58.40	
*3 + siblings*	100	26.67	
***Living locations***			
*Within Dhaka*	252	67.20	
*Outside of Dhaka*	69	18.40	
*Outside of Bangladesh*	54	14.40	

Note: MH (Mental health); SD (standard deviation).

### Prevalence of anxiety and depression

Out of 375 women in the sample, 59.2% reported symptoms of anxiety, including 6.4% with mild, 26.9% with moderate, and 25.9% with severe levels of anxiety. Similarly, 58.1% of participants reported depressive symptoms, including 5.3% with mild, 36.8% with moderate, and 16.0% with severe symptoms (Table 3). When outcomes were dichotomized into any anxiety (mild, moderate, or severe symptoms) versus no anxiety and any depression (mild, moderate, or severe symptoms) versus no depression, approximately 25.6% experienced both conditions, highlighting a substantial dual burden, while 33.6% reported anxiety only, 32.5% depression only, and 8.3% neither (Table 3).

**Table 3:** Levels of anxiety and depression among women.

*Levels of Anxiety*	*n*	*% (95% CI)*
*Minimal/none*	153	40.8 (35.8, 46.0)
*Mild*	24	6.4 (4.1, 9.4)
*Moderate*	101	26.9 (22.5, 31.7)
*Severe*	97	25.9 (21.5, 30.6)
***Depression***		
*Minimal/none*	157	41.9 (36.8, 47.0)
*Mild*	20	5.3 (3.3, 8.1)
*Moderate*	138	36.8 (31.9, 42.0)
*Severe*	60	16.0 (12.4, 20.1)
***Dual burden of anxiety and depression***		
*None*	31	8.3 (5.7 -11.5)
*Anxiety only*	126	33.6 (28.8 -38.60)
*Depression only*	122	32.5 (27.8 -37.5)
*Both*	96	25.6 (21.3 -30.30)

### Presenting MH symptoms

In Table 4, participants presenting MH symptoms in their first sessions were described. Distress, negative thinking, and feelings of worry were highly prevalent, reported by 90.54%, 87.39%, and 86.94% of women with anxiety, and by 79.82%, 99.54%, and 52.29% of those with mild to severe symptoms of depression, respectively. In contrast, trauma and self-harm behaviors were less commonly reported by 8.56% and 5.41% of those with anxiety, and by 14.22% and 5.96%of those with mild to severe symptoms of depression. Here it should be mentioned that percentages were calculated within each group (anxiety or depression). Percentages reflect women with a “Yes” response for each symptom category, stratified by anxiety and depression groups. The high percentages for symptoms such as distress, negative thinking and feelings of worry across both anxiety and depression groups likely reflect overlapping symptomatology between the two conditions.

**Table 4:** Presenting MH symptoms in women.

*Presenting MH symptoms*	*Anxiety*		*Depression*	
	Minimal/No	Mild to severe	Minimal/No	Mild to severe
***Distress***				
*No*	37.25	9.46	21.66	20.18
*Yes*	62.75	90.54	78.34	79.82
***Negative thinking***				
*No*	1.96	12.61	19.11	0.46
*Yes*	98.04	87.39	80.89	99.54
***Trauma***				
*No*	86.27	91.44	94.27	85.78
*Yes*	13.73	8.56	5.73	14.22
***Feelings of worry***				
*No*	81.70	13.06	31.85	47.71
*Yes*	18.30	86.94	68.15	52.29
***Self-harm***				
*No*	96.08	94.59	92.82	94.04
*Yes*	3.92	5.41	3.18	5.96

### Factors associated with anxiety

Table 5 presents the factors associated with anxiety among the participants. Women who completed their graduation had 66% lower likelihood of anxiety (AOR: 0.34; 95% CI: 0.15–0.79; p = 0.012) compared with women who are not graduates. Compared with those who had no siblings, having 1–2 siblings had a 50% lower likelihood of anxiety (AOR: 0.50; 95% CI: 0.25–0.99; p = 0.047), and having 3 or more siblings had a 54% lower likelihood of anxiety (AOR: 0.46; 95% CI: 0.22–0.98; p = 0.044).

**Table 5:** Factors associated with anxiety among women.

*Variables*	*AOR*	*SE*	*95% CI*	*p-values*
***Age***	0.98	0.02	0.95, 1.02	0.394
***Education***				
*Not graduate*	1			
*Graduate*	0.34	0.15	0.15, 0.79	0.012*
*Postgraduate*	0.66	0.28	0.29, 1.50	0.324
***Occupation***				
*Housewife*	1			
*Student*	0.86	0.36	0.37, 1.97	0.717
*Job holder*	0.75	0.24	0.39, 1.41	0.370
*Business*	0.46	0.30	0.12, 1.68	0.237
***Marital status***				
*Never married*	1			
*Currently married*	1.44	0.41	0.82, 2.51	0.204
*Formerly married*	0.50	0.27	0.17, 1.44	0.201
***History of MH condition***				
*No*				
*Yes*	1.73	0.54	0.94, 3.18	0.078
***Family history of MH condition***				
*No*	1			
*Yes*	1.55	0.70	0.64, 3.78	0.333
***Number of siblings***				
*Only child*	1			
*1-2 siblings*	0.50	0.17	0.25, 0.99	0.047*
*3 + siblings*	0.46	0.18	0.22, 0.98	0.044*
***Living locations***				
*Within Dhaka*	1			
*Outside of Dhaka*	0.74	0.22	0.41, 1.31	0.303
*Outside of Bangladesh*	0.53	0.18	0.27, 1.03	0.061
***Types of health conditions***				
***Chronic***				
*No*	1			
*Yes*	0.73	0.34	0.29, 1.84	0.506
***Gynecological***				
*No*	1			
*Yes*	2.11	1.47	0.54, 8.29	0.286
***Hormonal***				
*No*	1			
*Yes*	1.31	1.13	0.24, 7.07	0.75
***Others***				
*No*	1			
*Yes*	1.02	0.39	0.49, 2.16	0.95
***Constant***	8.96	7.91	1.59, 50.59	0.013

Note: MH (Mental health); AOR (adjusted odds ratio), SE (standard error), and CI (confidence interval), *p < 0.05, **p < 0.01.

***p < 0.001.

### Factors associated with depression among women

Table 6 presents factors associated with depression among women. Compared with women who had not graduated, graduate-level women had 3.41 times higher odds of depression (AOR = 3.41; 95% CI: 1.54–7.54; p = 0.003). Being a postgraduate had 2.63 times higher odds of depression (AOR = 2.63; 95% CI: 1.22–5.67; p = 0.014) compared with non-graduates. Women reporting hormonal illness had over 10 times higher odds of depression (AOR = 10.14; 95% CI: 1.06–97.07; p = 0.044) compared with those without such illnesses. Conversely, women with history of MH conditions had 52% lower odds of depression (AOR = 0.48; 95% CI: 0.27–0.87; p = 0.013), and those with other illnesses (migraine, allergy, or headache) had 65% lower odds (AOR = 0.35; 95% CI: 0.16–0.73; p = 0.005) compared with women without these conditions.

**Table 6:** Factors associated with depression among women.

*Variables*	*AOR*	*SE*	*95% CI*	*p-values*
***Age***	1.01	0.02	0.97, 1.05	0.653
***Education***				
*Not graduate*	1			
*Graduate*	3.41	1.38	1.54, 7.54	0.003**
*Postgraduate*	2.63	1.03	1.22, 5.67	0.014*
***Occupation***				
*Housewife*	1			
*Student*	1.03	0.43	0.45, 2.34	0.946
*Job holder*	0.75	0.24	0.40, 1.41	0.367
*Business*	1.08	0.72	0.29, 4.01	0.913
***Marital status***				
*Never married*	1			
*Currently married*	0.93	0.27	0.54, 1.63	0.811
*Formerly married*	2.06	1.18	0.67, 6.31	0.205
***History of MH condition***				
*No*	1			
*Yes*	0.48	0.14	0.27, 0.86	0.013*
***Family history of MH condition***				
*No*	1			
*Yes*	1.09	0.45	0.48, 2.46	0.843
***Number of siblings***				
*Only child*	1			
*1-2 siblings*	1.42	0.46	0.75, 2.68	0.283
*3 + siblings*	1.38	0.5	0.68, 2.82	0.375
***Living locations***				
*Within Dhaka*	1			
*Outside of Dhaka*	1.5	0.45	0.83, 2.72	0.178
*Outside of Bangladesh*	0.81	0.27	0.42, 1.55	0.519
***Types of health conditions***				
***Chronic***				
*No*	1			
*Yes*	1.23	0.57	0.50, 3.03	0.652
***Gynecological***				
*No*	1			
*Yes*	0.54	0.32	0.17, 1.75	0.305
***Hormonal***				
*No*	1			
*Yes*	10.14	11.69	1.06, 97.07	0.044*
***Others***				
*No*	1			
*Yes*	0.35	0.13	0.16, 0.73	0.005**
**Constant**	0.43	0.37	0.08, 2.36	0.329

Note: MH (Mental health); AOR (adjusted odds ratio), SE (standard error), and CI (confidence interval), *p < 0.05, **p < 0.01.

***p < 0.001.

## Discussion

Mental health conditions, particularly anxiety and depression, are among the leading health concerns affecting women globally, with a significant burden lying in low- and middle-income countries (LMICs) [44,48,52,53]. However, in such resource-constrained settings, the current availability of digital MH services, such as social media-based MH platforms and applications, has made significant strides toward increasing the accessibility of MH care among women [54,55]. Although online counseling services are widely available nowadays, evidence remains limited regarding how gender-specific information and the socio-demographic traits of online service consumers affect MH outcomes, particularly anxiety and depression. Therefore, this study aimed to explore the prevalence and associated factors of anxiety and depression of women actively accessing online MH counseling from WSIF (a pro-bono MH service platform). The study findings have the potential to support the identification of the population outreach gap and create the scope for strategizing MH condition-specific digital interventions for women.

As per our study findings, among the 375 women, the prevalence of anxiety symptoms was 59.2%, with 6.4% with mild, 26.9% with moderate, and 25.9% with severe levels of symptoms. Depressive symptoms were reported in 58.1% of participants, including 5.3% with mild, 36.8% with moderate, and 16.0% with severe symptoms. The dual burden of both conditions was prevalent among 25.6% of the women. Participants having siblings were less vulnerable to anxiety. Lower odds of depression were reported among women with a previous history MH illness and other medical conditions like migraine, allergy, or headache. Regardless of study sample size limitations, our study findings also reported that attainment of education level and hormonal illness were associated with mental health conditions among women.

### Prevalence of anxiety and depression

In our study, more than half of the participants were screened positive for anxiety (59.2%) and depression (58.1%), corroborating with other South Asian studies from Bangladesh, Nepal, India and Pakistan [47,49,56–58]. Such high rates reflect complex, overlapping gendered roles for women, encompassing household and occupational, reproductive, and care-giving responsibilities, influenced by social, physiological, and cultural determinants [59,60]. From discrimination in the employment sector, endless household expectations, economic dependency, to low decision-making autonomy, gender-based violence and cultural stigma; urban women experience intense layers of stressors which bidirectionally exacerbate their psychological burden and limit help-seeking tendencies [6,26,59]. To address these complex public health challenges, an integrated and focused intervention is required that provides social and financial protections, subsidizes community care-giving facilities, and creates gender equity across the public health and social development sectors.

### Dual burden of anxiety and depression

According to our study, approximately one in four women (25.6%) experienced both anxiety and depression, consistent with previous evidence that these disorders commonly coexist and share overlapping risk factors [61–64]. Our adjusted regression model showed lower odds of concurrent depression among women with anxiety. Given the substantial comorbidity observed in our study and the well-established literature, this finding may reflect the effects of adjustment in the multivariable model rather than a true inverse clinical relationship between anxiety and depression [61–65]. Several prior studies also indicated such comorbidities, leading to increased illness severity, chronicity, and impairment of psycho-social functions [62,66]. The coexistence of these conditions also multiplies the risks of suicidal attempts, failure of psychiatric management, and higher psychiatric hospitalization [62,67]. Therefore, timely and accurate screening, which distinguishes patients with "only anxiety," "only depression," and "both conditions," is essential for targeted interventions and informed allocation of resources. Moreover, consistent with global evidence, distress, negative thinking, and feelings of worry emerged as the most frequently reported symptoms among women experiencing anxiety and depression in our study. Prior research has shown that these symptoms commonly co-occur across internalizing disorders, particularly among women [68–70]. Studies from South Asian and other LMIC settings likewise report that tension, worry and negative thought patterns are core idioms of distress among women seeking mental health support [71,72]. Collectively, these findings underscore the importance of integrating cognitive-emotional regulation strategies, such as CBT-based and trans-diagnostic approaches, into digital mental health programs for Bangladeshi women [73,74].

### Mental health and social support

As per our study findings, women who had siblings were significantly less prone to anxiety. Similarly, prior studies found a strong positive correlation between sibling relationship quality and adult psychological well-being [41]. However, these findings should be interpreted within a broader socio-cultural context, as not all family structures provide the same level of emotional security. Consistently, studies have shown that warm, supportive sibling relationships, especially among sisters, are associated with reduced anxiety symptoms and greater life satisfaction [75]. A strong sibling bond was found to be protective for individuals against family-wide stressors and depressive symptoms, even during conflict-ridden times [76]. Similarly, numerous studies have highlighted the protective role of social support in shaping people’s coping abilities during psycho-social distress [45,77,78]. In developing country settings like Bangladesh, where MH resources are limited, social support, including sibling relationships, could be recognized as a vital informal network in psycho-intervention programs. In the context of digital MH services, individuals lacking family support can benefit from online peer support groups and digital communities. Digital MH support groups have been effective in reducing symptoms of both anxiety and depression [32,79]. Therefore, it is foreseeable that internet-based MH services could promote mental well-being by replicating the positive effects of familial and secure relationships.

### Mental health and previous history of MH conditions

Interestingly, our study reported that women with a history of MH conditions were significantly less likely have depressive symptoms. This is justified by several previous studies providing evidence that a previous history of MH counseling services has equipped them with coping skills and awareness that have reduced the odds-developing MH disorders [80,81]. Furthermore, abundant studies have evidenced the likely association between women with mental health literacy and lowered vulnerability to common mental health diseases [82–84]. Hence, women familiar with mental health services are more likely to recognize the early symptoms, increased help-seeking behavior and coping behaviors, lowering the risks of developing MH illness [12,21,26,84–86]. Several previous studies have shown that not only having a history of MH conditions but also shared pathway mechanisms through genetic susceptibility and stressful life events are linked to recurrent episodes of anxiety and depression [52,87,88]. Recognizing the potential outcomes regarding MH history and its influence on MH, online counseling services or peer group programs can prevent relapse and support long-term well-being.

### Mental health and other medical conditions

In our study findings, women with migraine, allergy, or headache reported lower likelihoods of developing depression. However, the majority of previous literature reports that migraine, allergy, and headaches are commonly associated with depression, and women with migraine show a significantly higher prevalence of depressive symptoms [89–92], specifically in women [92,93]. Although counter-intuitive, this finding can be partly explained by the role of social support and active health engagement among individuals managing these conditions, however, these mechanisms were not directly assessed in our study. Still, there is research supporting our findings that effective therapies for allergies and migraines reduce chronic inflammation, pain, sleep disturbances, and functional impairment, which contribute to lower likelihoods of depression [94,95]. Moreover, people undergoing treatments for such health conditions showed a lower risk of developing depression [94]. Prior research has also shown that stronger perceived social support is associated with lower depression levels in migraine patients and contributes to improved quality of life by enhancing pain self-efficacy and emotional resilience [96]. Collectively, these studies suggest that individuals managing these health conditions benefited from enhanced social and medical support, as well as treatment-related improvements in overall well-being, which could explain the observed inverse association with depression in our sample.

### Mental health and educational attainment

Our study findings interpreted that being a graduate was associated with lower odds of anxiety. On the other hand, graduate and postgraduate women were found to have higher odds of depression. Education is widely regarded as a resilience factor against MH conditions in numerous studies from LMIC settings [97–99]. However, some studies quoted that educated women may become emotionally vulnerable due to career and family conflict, organizational inequities, and limited advancement opportunities [97–99]. While our reported findings may be partially attributable to the study sample’s limitations and characteristics, policymakers should broaden their focus beyond education access to promote equitable, psycho-socially safe employment opportunities for women.

### Mental health and hormonal illness

While the limited sample size limits generalization, our study found that hormonal conditions are associated with MH of women participants of our study [100,101]. Previous studies, including a study from Iran, reported that patients with hormonal illnesses like hypothyroidism concurrently experience MH conditions [102]. Thyroid conditions are particularly associated with depressive symptoms, and treating thyroid conditions improves mood disturbances [103]. Hence, our findings warrant the need for further investigation of the role of hormonal conditions in deteriorating the mental well-being of women. Moreover, incorporating thyroid screening into digital MH platforms may be considered as part of an inclusive women’s mental healthcare approach.

Overall, the study findings created the scope for specific action plans integrating digital platforms for improved management of anxiety and depression among Bangladeshi women. As an immediate step, awareness-raising on common MH conditions like depression, anxiety, and stress can be implemented in a collaborative approach, and a referral pathway mechanism with organizations striving for women’s well-being. Additionally, this study can be utilized as one of the pivotal research-based pieces of evidence by policymakers and stakeholders to amplify and strengthen digital MH care platforms and online peer groups, providing accessible and sustainable MH services, especially during a public health crisis. The study’s results also give more scope and urge relevant stakeholders to assess nationwide women’s vital MH needs, disaggregated by service requirements, age, occupation, location, and MH conditions.

## Strengths and limitations

The cross-sectional design limits the ability to infer causal relationships between the observed associations. Self-reported presenting MH symptoms might be influenced by response or social desirability bias. Because WSIF is a mental-health support platform, our sample reflects women actively seeking MH support through online and mostly women with internet access, which may limit generalization. However, participants were reached via WSIF’ s public webpages and cross-platform awareness posts, not only from a closed group, and our analyses focus on associations among service-seeking women. Such association estimates are typically less sensitive to this kind of selection than population prevalence, though some selection-related bias cannot be entirely excluded.

Additionally, the study does not account for other relevant factors such as domestic violence, financial stress, or lifestyle factors like diet and physical activity, which may also affect MH outcomes. Furthermore, our study results cannot be generalized to the broader population, as the participants were already utilizing MH care services. Hence, these help-seeking groups of women are likely to exhibit increased mental health condition prevalence levels compared to the general population. However, our research study represents a significant and understudied group of women seeking digital mental health services and aims to identify their needs.

It is worth mentioning that, despite representing an educated, urban, and internet-connected population, our study revealed a high prevalence of depression and anxiety among women seeking MH care, comparable to facility-based and tele-psychiatry studies conducted in developing countries such as Pakistan, Nepal, SriLanka, and Ethiopia [17–19]. The coherence with global research further enhances the strength of our study and confirms the scope of high mental health outcomes among women with digital access. Our research findings highlight the multiple socio-demographic factors correlating with mental illnesses among the target group, strengthening the scope of the study.

## Conclusion

Aligning with the rising global mental health trends and initiatives, digital mental health services and their utilization have significantly increased among the Bangladeshi populations. However, the availability of abundant online mental health services does not validate the reliability and effectiveness of these initiatives. Considering this problem area, our study explored the prevalence and factors of anxiety and depression among the female users of digital MH care services. As per our study findings, the high anxiety and depression levels among female digital MH care users are correlated with past mental illness history, education level, sibling relationships and hormonal status. Our findings provide insights and scope for building tangible, need-based interventions for women. Moreover, this study can be used to state the need for future studies researching the needs of digital mental health services users.

### Implications of the study

This study offers novel insights into the potential of digital MH services as an alternative to traditional care in resource-limited settings. Additionally, the assessment of study participants was conducted using validated tools by MH professionals, thereby increasing assessment accuracy through the combination of a valid tool and professional judgment. The study’s findings on several potential socio-demographic variables enable a comprehensive understanding of factors contributing to anxiety and depression. Importantly, it identifies both associated factors (e.g., hormonal illnesses, higher education levels for depression) and protective factors (e.g., having siblings, a prior history of MH conditions), generating actionable insights for policymakers, MH professionals, and digital health developers. By focusing on women actively seeking online MH support, the study provides valuable evidence on patterns of digital MH service utilization, offering critical guidance for existing online mental health platforms in developing scalable, tailored, and gender-responsive digital interventions.

## Supporting information

S1 Table Health conditions of study participants. (DOCX)

S1 Checklist STROBE Checklist [CC BY 4.0 attribution]. (DOCX)
