# Supplementary material for: Prevalence and associated factors of anxiety and depression among women seeking digital mental health services in Bangladesh
Source: PLOS Ment Health. 2026 Sep 15;3(9):e0000715. doi: 10.1371/journal.pmen.0000715 (PMC13577547; doi:10.1371/journal.pmen.0000715)
Supplement: Supplementary file 1 [file pmen.0000715.s001.docx]

**S1 Table: Health conditions of study participants**

| **Categories** | **Definition** | **Health conditions** |
| --- | --- | --- |
| Chronic | Long-standing systemic conditions | Hypertension, diabetes, asthma, obesity, cancer, epilepsy |
| Gynaecological | Reproductive system-specific disorders | Gynaecological conditions, PCOS, menstruation-related conditions |
| Hormonal | Endocrine-driven conditions | Thyroid condition |
| Others | Common but distinctively different from the first three domains but are important contributors to women’s overall health burden | Migraine, Allergy, headache |
