# Supplementary material for: Prevalence and associated factors of anxiety and depression among women seeking digital mental health services in Bangladesh
Source: PLOS Ment Health. 2026 Sep 15;3(9):e0000715. doi: 10.1371/journal.pmen.0000715 (PMC13577547; doi:10.1371/journal.pmen.0000715)
Supplement: Supplementary file 2 [file pmen.0000715.s002.docx]

**S1 Checklist: STROBE Checklist** [CC BY 4.0 attribution]

|  | **Recommendation** | **Reported on Page** |
| --- | --- | --- |
| Title & Abstract | Indicate design and key results | 1 |
| Background/Rationale | Scientific background and rationale | 2-3 |
| Objectives | State specific objectives | 3 |
| Study Design | Present key elements of study design early | 2-5 |
| Setting | Describe setting, locations, relevant dates | 2-5 |
| Participants | Eligibility criteria, sources, methods | 2-5 |
| Variables | Clearly define all outcomes and predictors | 2-5 |
| Data Sources/Measurement | Sources and methods of assessment | 2-5 |
| Bias | Describe efforts to address potential bias | 13 |
| Study Size | Explain how sample size was determined | 2-5 |
| Quantitative Variables | Explain handling and grouping | 2-5 |
| Statistical Methods | Describe all statistical methods | 2-5 |
| Results | Participants, descriptive data, outcomes | 5-10 |
| Discussion | Key results, limitations, interpretation | 11-12 |
| Funding | Sources and role of funders | 15 |
